# Diagnostic Code Ambiguity and Misclassification of Adults With Spinal Muscular Atrophy: Single-Center Chart Review

**DOI:** 10.2196/103543

**Published:** 2026-07-28

**Authors:** Gabriel Holly, Bryce Bean, Haidy Beshay, Gabrielle Edwards, Nicholas Streicher

**Affiliations:** 1 Department of Neurology, School of Medicine, Georgetown University, 3800 Reservoir Rd., Washington, DC, United States, 1 202-877-1212; 2 MedStar Health Research Institute, Washington, DC, United States

**Keywords:** spinal muscular atrophy, ICD-10, clinical coding, International Classification of Diseases, electronic health records, rare diseases, disease-modifying therapy, natural language processing, cohort identification, SMN1, secondary use of clinical data, diagnostic miscoding

## Abstract

In a single US health system, fewer than one in three adults flagged by spinal muscular atrophy–associated diagnostic codes had molecularly confirmed spinal muscular atrophy (positive predictive value 27%), with the majority miscoded across clinically distinct categories.

## Introduction

Spinal muscular atrophy (SMA) is an autosomal recessive disorder caused by biallelic loss of the survival motor neuron 1 (SMN1) gene [1]. Since 2016, three disease-modifying therapies have been approved, all now available to adults. The antisense oligonucleotide nusinersen and the oral splicing modifier risdiplam benefit adults as well as children [2]. The gene-replacement therapy onasemnogene abeparvovec, previously limited to infants, reached patients aged 2 and older through an intrathecal formulation (2025). Adults now make up roughly half the prevalent population, an estimated 8000‐10,000 individuals [3]. Adults first diagnosed in childhood, when genetic confirmation was not routine and before treatment existed, may have had little reason to follow up, especially if mild or stable. Identifying them within a health system now relies on coded electronic health record (EHR) data.

The primary SMA diagnostic code is an unreliable marker. Diagnostic terminologies lack SMA-specific codes and group distinct disorders under shared rubrics, conflating true SMA with similarly named conditions such as spinal and bulbar muscular atrophy (SBMA) [4]. In a claims-based cohort, SMA codes did not distinguish subtypes or confirm true cases [5], and in a genomic cohort, overlapping codes could not confirm SMN1-SMA without genetic testing [6].

We quantified the positive predictive value (PPV) of SMA-associated diagnostic codes against chart adjudication in one US health system and characterized the miscoded cases.

## Methods

### Ethical Considerations

We conducted a single-center retrospective chart review in an academic multihospital health system in the mid-Atlantic United States, approved by the MedStar Health Research Institute Institutional Review Board (Protocol STUDY00008773) and conducted in accordance with the Declaration of Helsinki under a Health Insurance Portability and Accountability Act waiver.

### Study Design

On October 29, 2025, we identified all adults (≥18 y at the qualifying encounter) with any encounter in the preceding 10 years carrying an SMA-associated code in either *International Classification of Diseases, Tenth Revision, Clinical Modification* (*ICD-10-CM*; G12.0, infantile SMA, type 1; G12.1, other inherited SMA; G12.8, other SMA and related syndromes; G12.9, SMA, unspecified) or SNOMED CT (Systematized Nomenclature of Medicine Clinical Terms), since the EHR stores problem-list diagnoses in SNOMED CT, mapped to *ICD-10-CM* for billing. Each chart was attributed to the diagnostic code as entered by the provider, either *ICD-10-CM* or SNOMED CT. G12.0 was included because it can persist on adult charts through historical entries. Confirmed SMA required documented molecular SMN1 confirmation. Each chart, in which every patient had been evaluated by at least two neurologists, was reviewed for the documented diagnosis and responsible code, then classified as confirmed SMA, other appropriately coded motor neuron condition, SBMA, asymptomatic carrier, prenatal carrier screening, no relevant neuromuscular diagnosis, or unrelated. PPV was calculated with Wilson score 95% CIs. The per-code distribution, demographic characteristics, and category definitions appear in Multimedia Appendix 1.

## Results

Of 60 charts, 22 (37%, 95% CI 26%‐49%) were appropriately coded for SMA or a related motor neuron disorder. Sixteen (27%, 95% CI 17%‐39%) had molecularly confirmed SMA, and the other 6 were appropriately coded motor neuron conditions (5 Hirayama disease, 1 distal SMA). The PPV of an SMA-associated code for molecularly confirmed SMA was therefore 27%, rising to 37% if related motor neuron disorders are included. Among the 16 confirmed patients, all seen after 2016, 10 (63%) had no documented disease-modifying therapy.

The remaining 38 charts (63%) were miscoded (Table 1), comprising conditions unrelated to SMA (n=10, 17%), asymptomatic SMA carriers (n=9, 15%), charts with no relevant neuromuscular diagnosis (n=9, 15%), SBMA (n=5, 8%), and prenatal carrier screening (n=5, 8%).

**Table 1.** Adjudicated diagnosis of 60 adult charts flagged by spinal muscular atrophy (SMA)–associated diagnostic codes.

Adjudicated category	n	%
Confirmed SMA (molecularly confirmed)	16	27
Other appropriately coded motor neuron condition	6	10
Spinal and bulbar muscular atrophy	5	8
Asymptomatic SMA carrier	9	15
Prenatal carrier screening	5	8
No relevant neuromuscular diagnosis	9	15
Unrelated diagnosis	10	17
Total	60	100

## Discussion

Most adults whose chart carried an SMA code did not have SMA. Fewer than 1 in 3 had confirmed disease, well below the roughly 4 in 5 correct for the infantile SMA code in a national registry [7], the two-thirds for amyotrophic lateral sclerosis codes [8], and the one-half for muscular dystrophy [9]. No comparable estimate existed for the SMA-associated codes used in adults. Because the the ICD-10 codes provide no SMA subtype and bundle genetically distinct disorders, carriers, and screening encounters under shared rubrics, the code cannot by itself separate affected patients from unaffected ones.

Although SBMA and distal SMA can resemble SMA clinically, they are distinct genetic diseases. SBMA lacks a dedicated code and is often filed under SMA or adjacent codes, defensible administratively yet reflecting a classification *ICD-10* no longer captures. Asymptomatic carriers and prenatal screening encounters represent unaffected individuals. Counting any as SMA inflates the apparent cohort; here, miscoded charts outnumbered confirmed cases more than 2 to 1, so a registry built on these codes would be wrong before its first analysis. Because coding underlies disease queries, this undermines estimates of prevalence, treatment patterns, and natural history from single systems to national registries.

Correcting this requires two changes. First, the codes must be aligned with the clinical record so they are applied as documented and anchored on molecular SMN1 confirmation with type-specific subcodes so they are precise. Europe built Orphacodes, a dedicated rare-disease coding system, to capture diseases *ICD-10* cannot distinguish [10], and a comparable US reform through the National Center for Health Statistics could require SMN1 confirmation for the main SMA code, add subcodes for types 0 to 4, and retire the catchall categories. Second, these codes must then be applied consistently to every note, old and new. In future work, disease-specific natural language processing could recode existing records and check new entries against the documented diagnosis.

As a single-center, retrospective estimate from a small sample, this rate may not transfer to other settings, though the pattern appears in claims data elsewhere. Adjudication drew on records in which every patient had been evaluated by at least two neurologists, and confirmed cases were anchored on molecular testing rather than reviewer judgment. Because all confirmed patients were seen after 2016, undocumented therapy is not a pretreatment artifact of limited confirmation or stable disease; it more likely reflects care outside this system, which a single institution cannot distinguish from loss to follow-up. Privacy-preserving record linkage could close this gap.

Despite these limits, SMA-associated diagnostic codes are an unreliable marker for confirmed disease. Codes that are aligned, molecularly anchored, consistently applied, and audited would make identification reliable, strengthening prevalence estimates, treatment tracking, and natural-history research.

## Supplementary material

10.2196/103543 Multimedia Appendix 1 Supplementary tables.

## Abbreviations

EHR: electronic health record
*ICD-10-CM*: *International Classification of Diseases, Tenth Revision, Clinical Modification*
PPV: positive predictive value
SBMA: spinal and bulbar muscular atrophy
SMA: spinal muscular atrophy
SMN1: survival motor neuron 1
SNOMED CT: Systematized Nomenclature of Medicine Clinical Terms
