# Supplementary material for: Diagnostic Code Ambiguity and Misclassification of Adults With Spinal Muscular Atrophy: Single-Center Chart Review
Source: JMIR Med Inform. 2026 Jul 28;14:e103543. doi: 10.2196/103543 (PMC13412007; doi:10.2196/103543)
Supplement: Multimedia Appendix 1 [file medinform-v14-e103543-s001.docx]

**Multimedia Appendix 1. Supplementary tables.**

*Table A1. Appropriateness of coding by individual code.*

| **Code (terminology)** | **Appropriately coded** | **Miscoded** | **Total** |
| --- | --- | --- | --- |
| SNOMED 5262007 (spinal muscular atrophy) | 8 | 7 | 15 |
| SNOMED 128212001 (SMA type 2) | 4 | 0 | 4 |
| SNOMED 230247001 (distal SMA) | 1 | 0 | 1 |
| ICD-10 G12.0 (infantile SMA, type 1) | 0 | 1 | 1 |
| ICD-10 G12.1 (other inherited SMA) | 3 | 10 | 13 |
| ICD-10 G12.8 (other SMA and related syndromes) | 5 | 1 | 6 |
| ICD-10 G12.9 (SMA, unspecified) | 1 | 19 | 20 |
| **Total** | **22** | **38** | **60** |

*Table A2. Demographics of the 16 molecularly confirmed adults.*

| **Characteristic** | **Value** |
| --- | --- |
| Median age, years (range) | 36 (19–69) |
| Female, n | 8 |
| Male, n | 8 |

*Table A3. Adjudication categories.*

| **Category** | **Definition** |
| --- | --- |
| Confirmed SMA | Documented molecular SMN1 confirmation. |
| Other appropriately coded motor neuron condition | A distinct motor neuron disorder correctly captured by the assigned code (Hirayama disease under G12.8; distal SMA under SNOMED 230247001). |
| Spinal and bulbar muscular atrophy (SBMA) | Genetically distinct from SMA, with no dedicated code, so it is frequently, though not only, coded under SMA codes. |
| Asymptomatic SMA carrier | Documented heterozygous SMN1 carrier without disease, identified outside a reproductive-screening context. |
| Prenatal carrier screening | Code entered during preconception or prenatal reproductive screening. |
| No relevant neuromuscular diagnosis | No documented neuromuscular diagnosis supporting the code. |
| Unrelated diagnosis | A documented condition unrelated to SMA or motor neuron disease. |
